# Prevalence of Metabolic Syndrome Based on Activity Type and Dietary Habits in Extremely Low-Income Individuals

**DOI:** 10.3390/nu16111677

**Published:** 2024-05-29

**Authors:** Kunxia Su, Yonghwan Kim, Yoonjung Park

**Affiliations:** 1 Department of Sports, Henan University of Chinese Medicine, Zhengzhou 450046, China; skxlyn1314@163.com; 2 Department of Physical Education, Gangneung-Wonju National University, Gangneung 25457, Republic of Korea; 3 Laboratory of Integrated Physiology, Department of Health and Human Performance, University of Houston, Houston, TX 77204, USA

**Keywords:** activity type, basic livelihood security, dietary habit, socioeconomic status

## Abstract

A high prevalence of metabolic syndrome (MS) and cardiovascular disease among low-income individuals has often been reported. However, there is still a lack of research on the relationship between basic livelihood security (BLS) and MS. This study investigated the prevalence of MS according to activity type, dietary habits, and the nutrient intake characteristics of individuals receiving BLS. Data from 14,803 men and 20,299 women were analyzed to assess the association between receiving BLS and MS. The associations between MS and various factors were analyzed separately in men and women by logistic regression analysis. In this cohort, 5.9% of men and 6.8% of women received BLS; of these, 46.9% and 47.7% had MS, respectively. High caloric intake, low-frequency breakfast consumption, and no nutritional education were associated with MS in both men and women. Among those with a low-frequency walking habit and strength training activity type, MS increased by 1.58 and 1.57 times in men and by 1.47 and 2.16 times in women, respectively. Men who were sedentary for 8 h or more had an increased risk of MS, but there was no association between these in women. BLS nutritional intake characteristics were high in carbohydrates and fat and low in dietary fiber and vitamin C (*p* < 0.05). In conclusion, establishing a healthy eating pattern through nutritional education and increasing walking and strength training may reduce the risk of MS.

## 1. Introduction

Metabolic syndrome (MS), which can cause coronary artery disease or stroke, refers to a cluster of cardiovascular disease factors and is characterized by various risk factors such as increased lipids, blood pressure, and obesity [1]. MS in Korean adults steadily increased from 27.1% in 2001 to 33.2% in 2020 [2]. This trend is similar in the United States. MS increased from 37.6% in 2011 to 41.8% in 2018 [3]. MS has become a particular concern, however, because MS does not have symptoms such as pain, patients may not seek care until life-threatening symptoms such as angina pectoris, myocardial infarction, or stroke occur. Once symptoms develop or it becomes fatal, personal and social costs increase. Additionally, MS has a negative impact on the quality of life of individuals and families [4,5]. Therefore, the World Health Organization (WHO), governments, and health officials worldwide are making efforts toward early management of MS [6].

Direct factors that contribute to MS include age, smoking, alcohol consumption, lack of activity type, and dietary habits. Social factors such as education, personal economic status, residential area, and divorce are also known to have a negative effect as indirect factors [7]. A previous meta-analysis in France reported that the prevalence of MS increased 1.04 to 1.16 times in individuals with low socioeconomic status (SES) [8,9]. It is assumed that low SES results in reduced financial ability, opportunity, and time to manage the disease; further, these individuals tend to have lower levels of awareness of the disease and lower trust in treatment [9]. Therefore, government officials planning welfare policies are trying to provide welfare to allow a minimum standard of living to people in extremely difficult economic conditions. The South Korean government is also making various efforts to provide support to those in extremely low-income situations, termed basic livelihood security (BLS) [10,11].

This system has been operating a system called Public Charge or Social Security for a long time not only in Korea but also in developed countries such as the United States [12]. The government describes this as “a system that guarantees a minimum standard of living and promotes self-reliance by providing necessary benefits to people who have difficulty making a living” [13]. The services provided include a social security system that provides tax breaks, economic support, housing, and medical care. Moreover, BLS considers not only income but also age, health, and family members who have no or extremely low income or low income potential [13]. Research to understand the characteristics of individuals receiving BLS is still in progress from various perspectives, and one of the most notable features is that receiving BLS is associated with a high prevalence of various diseases. For example, individuals receiving BLS have a 33.1% higher risk of osteoporosis than individuals not receiving BLS [14]. The proportion of people receiving BLS with depression is relatively high (10.1%), and accessibility to hospitals due to depression and sensitivity in recognizing one’s own health is low [15]. In a cohort study targeting residents living in Lausanne, Switzerland, it was found that MS was 1.47 times higher in the low-income group compared to high-income earners [16]. In addition, it was said that there was a significant difference in nutritional intake depending on household income. Valsta et al. reported that low-income men had low dietary fiber intake and women had high carbohydrate and low fat intake [17].

Although the health status of people with extremely low incomes, such as those receiving BLS, should be fully investigated, there is still a lack of research on the relationship between receiving BLS and MS [18,19]. Therefore, the purpose of this study was to compare the prevalence of MS in individuals receiving and not receiving BLS and to identify MS characteristics according to activity type and dietary habits in individuals receiving BLS. Such research would be especially meaningful in understanding health habits and working toward reducing the risk of MS. Furthermore, the characteristics of dietary nutrient intake in BLS were confirmed.

## 2. Materials and Methods

### 2.1. Data and Study Population

This study used data from a national survey conducted by the government. Men and women aged 30 years or older between 2014 and 2022 were included in the study (total 35,102; 14,803 men and 20,299 women). Individuals who did and did not receive BLS were included after excluding those with missing data and those who did not undergo testing relevant to this study. Individuals who indicated in the questionnaire that they had previously or were currently receiving BLS were included in the BLS group; individuals who had not received BLS were selected for the non-BLS group. The main findings of this study, which were BLS status, physical activity (type, frequency, amount), and dietary habits, were assessed using a questionnaire. Meanwhile, MS elements (blood pressure, blood collection, waist circumference) were physically measured. Survey details, from a questionnaire, included past and present medical history, drug history, and social factors such as education, income, employment, and marital status. Alcohol consumption and smoking status were included as health behaviors, and activity type and dietary habits were included in the major analysis.

### 2.2. Metabolic Syndrome

The diagnosis of MS was based on the criteria provided by the third report of the National Cholesterol Education Program [20] and waist circumference, which indicates abdominal obesity, was determined according to the WHO Asia Pacific guidelines [21]. Individuals who met three or more of the following five criteria were diagnosed with MS: (1) waist circumference ≥90 cm for men and ≥85 cm for women; (2) triglycerides ≥150 mg/dL; (3) fasting blood sugar level ≥100 mg/dL; (4) high-density lipoprotein cholesterol <40 mg/dL for men and <50 mg/dL for women; and (5) blood pressure ≥130/85 mmHg. Individuals diagnosed with high blood pressure, diabetes, or dyslipidemia, and those who were taking medications, were considered to have additional risk factors.

### 2.3. Activity Type

Activity type was based on the activity questionnaire produced and distributed by the WHO [22]. Activity type was classified in detail based on the intentions of the survey organization. During work and leisure, moderate to high activity (MHA), strength training, walking frequency, and sedentary time were investigated. Walking frequency was divided into three groups: 6–7 days, 3–5 days, and 0–2 days. The strength training groups were 4–7 days, 2–3 days, and 0–1 day. MHA was written as a subjective judgment and was investigated through work and leisure. Lastly, sedentary lifestyle was divided into two groups based on 8 h per day.

### 2.4. Dietary Pattern

A food frequency questionnaire was developed by the survey organization, and its reliability and validity were verified [23,24]. The survey was conducted using a 24 h recall method, and daily caloric intake was calculated (Supplementary Materials, Table S1). The questionnaire included weekly breakfast intake frequency, eating out frequency, experience with nutrition education, awareness of the nutrition contained in food (‘Do you eat with awareness of nutritional information?’), and subjective state (‘Have you ever not been able to eat enough due to financial difficulties?’).

### 2.5. Data Analysis

Statistical analyses were performed using SPSS software (version 25.0; SPSS Inc., Chicago, IL, USA). Differences in MS were assessed between men and women in the BLS and non-BLS groups. Continuous variables are expressed as means and standard deviations, and comparisons between groups were performed using an independent *t*-test. Categorical variables are expressed as numbers and percentages and were analyzed using the chi-square test. Sociodemographic characteristics were compared between the BLS and non-BLS groups and further divided into subgroups by sex. Multiple regression analysis was performed using these variables, and adjustment variables for the logistic regression analysis were identified. The odds ratio (OR) and 95% confidence interval (CI) for MS prevalence were calculated using multivariate logistic regression analysis according to activity type and dietary patterns. The standard for non-BLS was G1 (group 1); for the BLS group, G1 of the non-BLS group was used as the reference group. *p* < 0.05 was defined as statistically significant.

## 3. Results

### 3.1. Participant Characteristics

Both men and women in the BLS group were older than those in the non-BLS group. Additionally, participants in the BLS group had significantly elevated systolic blood pressure and triglyceride and blood sugar levels compared to those in the non-BLS group. However, there were no notable differences in waist circumference among men or in diastolic blood pressure among women (Table 1).

### 3.2. Sociodemographic Characteristics

The BLS group had fewer individuals with college education or higher than the non-BLS group, with only 14.6% of men and 10.1% of women in the BLS group attending college. Compared to the non-BLS group, the BLS group had higher levels of unemployment, divorce, current smoking habits, and alcohol consumption. MS was 46.9% in men and 47.4% in women in the BLS group, showing significant disparities compared with the non-BLS group. When comparing men and women in the BLS group, significant differences were found in age, educational level, marital status, smoking, and alcohol consumption, whereas MS prevalence, occupation, and residence did not exhibit significant differences (Table 2).

### 3.3. Multiple Regression Analysis and Metabolic Syndrome

Multiple regression analysis was performed for men and women to determine which variables should be adjusted in the logistic regression analysis. For men, age, education level, occupation, smoking, and alcohol consumption were significant factors; for women, age, education, occupation, residence, smoking, and alcohol consumption, but not marital status, were significant factors (Table 3).

### 3.4. Association of Metabolic Syndrome and Dietary Pattern

The results of the logistic regression analysis for dietary pattern are presented in Table 4. In both men and women in the non-BLS group, caloric intake, breakfast frequency, eating out, nutritional education, and nutritional awareness were significantly associated with MS. In men in the BLS group, the ORs for MS were 1.20 for a caloric intake of reference dietary intake (RDI) or more, 1.24 for low-frequency breakfast, and 1.11 and 1.30 for nutritional education and awareness, respectively. In women in the BLS group, the corresponding ORs were 1.31, 1.11, 1.20, and 1.35, respectively. Among individuals with insufficient dietary habits, the incidence of MS decreased by 6% (Table 4).

### 3.5. Association between Metabolic Syndrome and Activity Type

**Figure 1:**
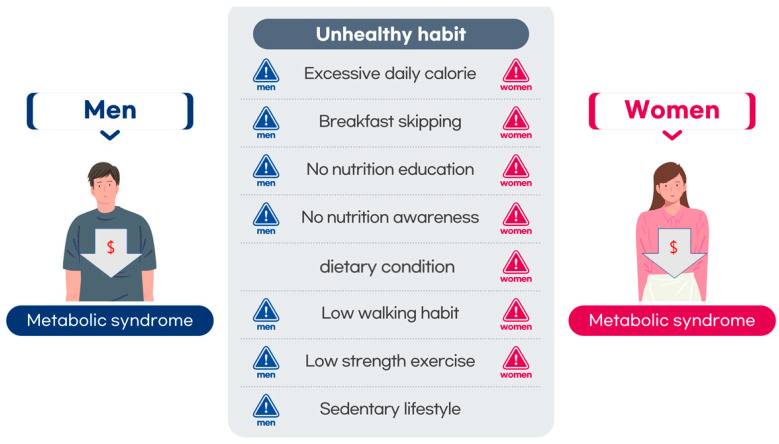
Significant health habits and metabolic syndrome in BLS men and women.

The results of the logistic regression analysis for activity type are presented in Table 5 and Figure 1. In people with a low frequency of walking and strength training, the risk of MS increased from 1.41 to 2.16 times in all groups. There was no significant difference in work-related MHA among the groups, and leisure MHA was significantly associated with MS only in the non-BLS group. There was no significant difference in the risk of MS based on leisure MHA in the BLS group. MS increased 1.31 times in the BLS group for men who worked sedentarily for more than 8 h, but there was no significant difference for women.

### 3.6. Comparison of Nutrient Intake between Groups

For comparison, under the same non-BLS and BLS conditions, a common denominator was created and normalized and nutrients were compared between groups (Table 6). The results are the same for men and women: compared to non-BLS, the BLS group had significantly higher intakes of carbohydrates, fat, cholesterol, and natrium, and significantly lower intakes of protein, dietary fiber, vitamin C, and unsaturated fatty acids (*p* < 0.05). Meanwhile, saturated fatty acids were not significant between groups in men (*p* > 0.05), and BLS was significantly lower in women (*p* < 0.05).

## 4. Discussion

Previous studies have examined MS in individuals with low SES, those with low income, and vulnerable populations [8,25]. However, this study investigated the relationship between dietary patterns, physical activity, and MS in extremely low-income individuals who require government support. We aimed to examine the impact on the healthcare and health behavior of people with economic difficulties.

In this study, the prevalence of MS in both men and women in the BLS group was significantly higher than that in the non-BLS group. These results are consistent with those of previous studies [26,27,28]. A cross-sectional study conducted in China surveyed 3175 adults aged 45 years or older and found that the incidences of MS were 65.4% in those with an income of less than 2000 yuan and 3.0% in those with an income of more than 6000 yuan [26]. In a 10-year longitudinal follow-up study in the United Kingdom, the lowest income group had a 1.27 times higher mortality risk (hazard ratio) due to heart disease than the highest income group and a 1.27 times higher risk ratio for all causes of mortality [27,28]. However, research on economic status and disease prevalence remains controversial. Rahman et al. retrospectively analyzed data from the United States and confirmed the association between SES and the prevalence of hypertension, hyperlipidemia, depression, cancer, and heart disease. The results showed that employment status, hyperlipidemia, chronic obstructive pulmonary disease, and marital status had significant effects on hypertension and hyperlipidemia; however, annual income had no significant effect in any group [28]. Experts have highlighted the importance of carefully monitoring the health awareness of low-income individuals, as they have fewer opportunities to visit hospitals and face financial difficulties in receiving treatment for diseases [29]. A study by Lazar et al. [30] in the US stated that low-income people have health insurance problems, a lack of health education, and a tendency to distrust medical service providers, which makes them hesitant to choose appropriate medical services. Therefore, in addition to providing cost benefits to BLS recipients, awareness of the health system should be improved at the same time.

Several studies have indicated that eating out has negative effects on health [31,32]. Gesteiro et al. found that a high rate of eating out reduces diet quality, including increased caloric, saturated fat, sugar, and sodium intake and lower intake of fiber, dairy products, and vegetables [31]. In this study, the OR for MS for eating out frequency was significant in the non-BLS group, however, it was not significant in the BLS group. This may be because participants could not afford to eat out due to financial difficulties, or they may have been eating out mainly for low-priced meals or simple meals.

We found a 1.20-fold increase in MS among women without nutritional education in the BLS group and a 1.35-fold increase in MS in women without nutritional awareness. These results are consistent with those of previous studies [33,34]. In a Korean cross-sectional study investigating the prevalence of MS in patients with cancer, the prevalence of MS in patients who did not receive nutritional education increased 1.28-fold, and in individuals without cancer, it also increased 1.36-fold [33]. In an experimental study conducted in Kenya, MS improved by 86 to 69% in the nutritional education group, while MS tended to worsen from 88 to 91% in the standard treatment group without nutritional education [34]. Therefore, nutritional education and awareness must be improved. Nutritional education includes nutrient interpretation, cooking methods and practices, and food product selection, and it can be easily conducted at the school level [35]. However, a decline in various educational opportunities due to the low educational levels of individuals with low incomes may result in decreased nutritional education. We also found that the educational level in the BLS group was very low. Therefore, for adults who are already past school age, education based on local communities, the internet, or mobile devices is required. A 3-month experimental study in adults with MS found that in-depth nutritional and dietary education over the phone decreased body weight and abdominal circumference and reduced the prevalence of MS to 24% lower than that in the control group [36]. In addition, a 6-month experimental study showed a significant reduction in total cholesterol in the nutritional education group with practical training compared to the control group [37]. A longer follow-up study of 3.6 months found that the prevalence of MS was 16% lower in the group that received community-based nutritional education [38]. Therefore, inducing changes in nutrient awareness and dietary patterns through nutritional education in individuals receiving BLS is necessary.

Unsurprisingly, we found that caloric intake, both high and low, was associated with the risk of MS. In those who consumed calories above the RDI, there was an increase in MS in the BLS group (ORs of 1.20 for men and 1.30 for women), and the result was similar in the non-BLS group. We also asked about subjective dietary status, specifically about not consuming enough food due to economic reasons. A 6% decrease in MS was observed in women in the BLS group with insufficient dietary conditions. This may be due not only to low caloric intake but also to overall unbalanced nutrition. Malnutrition is associated with a low risk of obesity, but it increases the risk of coronary artery disease and heart failure [39]. Therefore, monitoring BLS, which is associated with nutritional vulnerability, can help to improve nutritional status, which is the basis for health.

Previous studies have shown a strong relationship between breakfast frequency and MS, with the main hypothesis that skipping breakfast can cause MS. In a cross-sectional study conducted in Japan by Katsuura-Kamano et al., the group that skipped breakfast had a 1.26-fold increase in MS compared to the group that had breakfast every day [40]. Our results were similar, with the incidence of MS increasing by 1.10 to 1.24 times in all groups in individuals who skipped breakfast. There are various reasons for skipping breakfast, including overeating on the previous day, poor physical condition, busy morning hours, and the desire to reduce daily calorie intake. Therefore, some scholars have positive opinions about skipping breakfast and state that there is no need for guilt in relation to it [41].

Low activity and a sedentary lifestyle have long been considered typical precipitating factors of MS [42]. Our results showed that the risk of MS increased among those with a low frequency of walking and strength exercises, which is consistent with existing knowledge. Physical activity directly oxidizes carbohydrates and fats into energy, preventing fat accumulation in the body and increasing insulin sensitivity, thus making it easier to control blood sugar levels. In addition, it prevents an increase in blood pressure by increasing the elasticity of blood vessels [43]. Previous studies on MHA have also claimed that it prevents cardiovascular risk factors. A large study conducted in the United States found that low MHA was associated with a 12%, 4%, and 6% higher risk of diabetes, low high-density lipoprotein, and high triglyceride levels, respectively [44]. Additionally, a cross-sectional study showed that MHA could reduce the risk of MS by 21–33% and that increased walking could reduce the prevalence of MS by 35–55% [45]. The results of this study were consistent in the non-BLS group but not in the BLS group; there was no significant association between work-related MHA and MS in any group. In addition, considering that the unemployment rate was approximately 60% in the BLS group, it is difficult to confirm the results of this study. It is possible that many of these patients were excluded. A more detailed investigation of intensity and activity during work should be conducted.

The last major outcome of this study was a comparison of nutrients based on intake of BLS and non-BLS foods. While the non-BLS group had higher intake of unsaturated fat, dietary fiber, and vitamin C, they also had higher intake of carbohydrates, cholesterol, and vitamin C. These results are similar to previous results. A study in Germany found that very-low-income people consumed high fat, potatoes, and meat, while high-income people consumed low fat and dietary fiber and high vegetable protein [46]. A Finnish study found that low-income groups had lower dietary fiber intake and higher carbohydrate and fat intake [17]. Low-income people are bound to have limited food choices, and limited foods inevitably lead to nutritional imbalance. Supporting this fact, an American study found that people with low SES had lower food purchasing costs and reported purchasing less protein, dietary fiber, and vegetables for the same amount of money [47]. 

One of the strengths of this study is that men and women were analyzed separately. A Chinese study surveyed individuals over the age of 20 years in 31 regions and found a significant difference in the incidence of MS based on sex (30.0% in men and 32.3% in women) [48]. Mortality due to cardiovascular disease is higher in men in Asia [49]; conversely, in Europe, it is higher in women [50]. Sex differences in diseases can be caused by various factors, such as physiological factors, socioeconomic environment, cultural factors, regional and racial characteristics, and customs; additionally, obtaining healthcare may be more difficult for women than for men [51]. In a cross-sectional survey of more than 10,000 Chinese participants, the prevalence of MS according to SES was analyzed. Even after adjusting for age, smoking, alcohol consumption, activity type, and body mass index, the prevalence of MS was 6% lower for men and 28% lower for women in those in high-income households than for those in low-income households. Compared with low-income earners, the prevalence of MS also decreased by 6% in women with median income, although it was not significant for men [52]. In this study, the prevalence of MS was slightly higher in women than in men, although this difference was not statistically significant.

In South Korea, an income of 35% of the standard median income is considered to be a basic living income; therefore, the government is building a system to resolve various inequalities. The basic income standard differs every year, and the proportion of recipients of the National BLS is generally maintained at 2–3% of the total population [10]. As recipients of BLS experience many diseases and inequalities, various policies are being implemented to compensate, and medical, education, and living expenses are supported. However, the problem with BLS is not simple. Although many studies have noted the lower health status of individuals receiving BLS, they are also less likely to have high-quality education, advance to higher levels of education, and have high-salary jobs, and are more likely to live in areas with low access to healthcare and have a lower frequency of medical visits due to lower income, services, the burden of expensive treatment, and low disease awareness [15,30]. We observed similar findings. Individuals in the BLS group were older, more likely to be unemployed, had a higher divorce rate, were more rural, and were more likely to be current smokers.

Unfortunately, this phenomenon also applies to children and adolescents. Among adolescents belonging to these households, those with household food insecurity had a 1.38 times higher incidence of MS, and the proportion of individuals with a household income below the poverty level was high [53]. In another study of adolescents aged 13–18 years, MS not only increased 1.5 times in males and 4 times in females in low-income groups, but they also had a high rate of smoking and alcohol consumption [54].

The results of this study will be very useful for practical applications. Healthcare experts from medical, government, and public health organizations responsible for BLS can provide a deeper background in understanding their characteristics. One outcome for which BLS differs from non-BLS is the lack of nutritional education in men. In addition, while the intake of carbohydrates, cholesterol, and natrium is high in men and women with BLS, the intake of unsaturated fatty acids, dietary fiber, and vitamin C, which are known to be beneficial to health, is relatively low. Therefore, in order to prevent or improve MS, efforts should be made to increase activity as well as education on low-cost dietary habits and food selection.

This study has several limitations. This is a cross-sectional study; therefore, it is difficult to determine the effect of economic status on the cause of the disease. In addition, people who are currently extremely low-income but in welfare-blind areas may not have been selected by the BLS.

## 5. Conclusions

MS was more common in the BLS group than in the non-BLS group. In the non-BLS and BLS groups, an increased prevalence of MS was associated with high caloric intake, low breakfast frequency, low nutritional awareness, low walking frequency, and low strength exercise. In the BLS group, MS was associated with not receiving nutritional education, whereas in the non-BLS group it was associated with the frequency of eating out and low MHA leisure activities. High BLS and a sedentary lifestyle in men increased the risk of MS. Men and women with BLS had higher carbohydrate, cholesterol, and natrium intakes, while their intakes of unsaturated fatty acids, dietary fiber, and vitamin C were lower than non-BLS. Establishing an appropriate eating culture and increasing activity type through nutritional education in the BLS group may reduce the risk of MS.

## Figures and Tables

**Table 1:** Metabolic syndrome factors and general information on participants (n = 35,102).

Variables	Men		*p*	Women		*p*
	Non-BLS (n = 13,926, 94.1%)	BLS (n = 877, 5.9%)		Non-BLS (n = 18,911, 93.2%)	BLS (n = 1388, 6.8%)	
Age, years	55.6 ± 14.2	60.5 ± 13.2	<0.001	54.5 ± 13.8	61.2 ± 13.6	<0.001
Waistline, cm	88.8 ± 8.8	90.2 ± 10.0	0.177	82.4 ± 9.6	85.7 ± 10.4	<0.001
Systolic blood pressure, mmHg	122.1 ± 15.1	124.0 ± 16.7	<0.001	118.1 ± 17.6	123.3 ± 18.3	<0.001
Diastolic blood pressure, mmHg	77.7 ± 10.3	76.1 ± 10.9	<0.001	73.8 ± 9.4	74.1 ± 9.8	0.244
Triglyceride, mg/dL	159.6 ± 126.9	172 ± 158.3	0.006	116.1 ± 78	128.3 ± 79.6	<0.001
HDLC, mg/dL	47.5 ± 11.6	46.6 ± 12.2	0.019	55.2 ± 13.2	52.5 ± 12.7	<0.001
Glucose, mg/dL	105.8 ± 25.6	112.5 ± 36.4	<0.001	99.6 ± 21.5	105.1 ± 27.9	<0.001
Income, thousand Won	4471.3 ± 3229.2	1675.6 ± 1678.5	<0.001	4361.5 ± 3261.1	1683.6 ± 1705.8	<0.001
MS, n (%)	5684 (40.8%)	411 (46.9%)	<0.001	5947 (31.4%)	658 (47.7%)	<0.001
MS waistline, n (%)	6378 (45.8%)	430 (49.0%)	0.175	5743 (30.4%)	625 (45.0%)	<0.001
MS blood pressure, n (%)	7253 (52.1%)	525 (59.9%)	<0.001	7420 (39.2%)	780 (56.2%)	<0.001
MS triglyceride, n (%)	6657 (47.8%)	445 (50.7%)	0.091	6562 (34.7%)	621 (44.7%)	<0.001
MS HDLC, n (%)	3561 (25.6%)	277 (31.6%)	<0.001	6863 (36.3%)	616 (44.4%)	<0.001
MS glucose, n (%)	6976 (50.1%)	508 (57.9%)	<0.001	6487 (34.3%)	634 (45.7%)	<0.001

*p* < 0.05; BLS, basic livelihood security; non-BLS, non-basic livelihood security; MS, metabolic syndrome; HDLC, high density lipoprotein cholesterol.

**Table 2:** Demographic profile of participants (n = 35,102).

Category	Men		Women		Sex, *p* ^a and b^
	Non-BLS (n = 13,926, 94.1%)	BLS (n = 877, 5.9%)	Non-BLS (n = 18,911, 93.2%)	BLS (n = 1388, 6.8%)	
MS, n (%)	*p* < 0.001		*p* < 0.001		
Non-MS	8242 (59.2%)	466 (53.1%)	12,964 (68.6%)	730 (52.6%)	^a^ *p* < 0.001 ^b^ *p* = 0.829
MS	5684 (40.8%)	411 (46.9%)	5947 (31.4%)	658 (47.4%)	
Age Group	*p* < 0.001		*p* < 0.001		
30–39	2398 (17.2%)	65 (7.4%)	3354 (17.7%)	108 (7.8%)	^a^ *p* < 0.001 ^b^ *p* = 0.033
40–49	2749 (19.7%)	126 (14.4%)	4097 (21.7%)	220 (15.9%)	
50–59	2891 (20.8%)	206 (23.5%)	4296 (22.7%)	256 (18.4%)	
60–69	3100 (22.3%)	221 (25.2%)	3970 (21.0%)	338 (24.4%)	
70–80	2891 (20.8%)	206 (23.5%)	3194 (16.9%)	466 (33.6%)	
BMI	*p* < 0.001		*p* < 0.001		
≤22.9	5431 (39.0%)	270 (30.8%)	8893 (47.0%)	515 (37.1%)	^a^ *p* < 0.001 ^b^ *p* = 0.205
23.0–24.9	3259 (23.4%)	237 (27.0%)	4171 (22.1%)	300 (21.6%)	
≥25.0	5239 (37.6%)	370 (42.2%)	5839 (30.9%)	573 (41.3%)	
Education Status	*p* < 0.001		*p* < 0.001		
To elementary	2068 (14.8%)	347 (39.6%)	4581 (24.2%)	699 (50.4%)	^a^ *p* < 0.001 ^b^ *p* < 0.001
To middle	1545 (11.1%)	159 (18.1%)	2040 (10.8%)	197 (14.2%)	
To high	4282 (30.7%)	243 (27.7%)	5709 (30.2%)	352 (25.4%)	
Above college	6031 (43.3%)	128 (14.6%)	6581 (34.8%)	140 (10.1%)	
Occupation	*p* < 0.001		*p* < 0.001		
Yes	10,575 (75.9%)	361 (41.2%)	9896 (52.3%)	539 (38.8%)	^a^ *p* < 0.001 ^b^ *p* = 0.271
No	3351 (24.1%)	516 (58.8%)	9015 (47.7%)	849 (61.2%)	
Marital Status	*p* < 0.001		*p* < 0.001		
With spouse	11,768 (84.5%)	472 (53.8%)	14,730 (77.9%)	537 (38.7%)	^a^ *p* < 0.001 ^b^ *p* < 0.001
Not married	1319 (9.5%)	166 (18.9%)	897 (4.7%)	81 (5.8%)	
Death of spouse	315 (2.3%)	57 (6.5%)	2417 (12.8%)	474 (34.1%)	
Divorce	524 (3.8%)	182 (20.8%)	867 (4.6%)	296 (21.3%)	
Residence Region	*p* = 0.015		*p* < 0.001		
City	10,905 (78.3%)	656 (74.8%)	15,260 (80.7%)	1063 (76.6%)	^a^ *p* < 0.001 ^b^ *p* = 0.338
Rural	3021 (21.7%)	221 (25.2%)	3651 (19.3%)	325 (23.4%)	
Smoking History	*p* < 0.001		*p* < 0.001		
Never	2882 (20.7%)	150 (17.1%)	17,141 (90.6%)	1129 (81.3%)	^a^ *p* < 0.001 ^b^ *p* < 0.001
Past	6705 (48.1%)	369 (42.1%)	1050 (5.6%)	112 (8.1%)	
Current	4339 (31.2%)	358 (40.8%)	720 (3.8%)	147 (10.6%)	
Alcohol Frequency	*p* < 0.001		*p* < 0.001		
None or 1–2 per month	5481 (39.4%)	455 (51.9%)	13,573 (71.8%)	1086 (78.2%)	^a^ *p* < 0.001 ^b^ *p* < 0.001
1 per week	3430 (24.6%)	130 (14.8%)	3314 (17.5%)	162 (11.7%)	
>2 per week	5015 (36.0%)	292 (33.3%)	2024 (10.7%)	140 (10.1%)	

BLS, basic livelihood security. ^a^, men non-BLS vs. women non-BLS; ^b^, men BLS vs. women BLS.

**Table 3:** Multiple regression analysis and metabolic syndrome.

Variables	Men					Women				
	B	SE B	*ß*	*t*	*p*	B	SE B	*ß*	*t*	*p*
Age	0.004	0.000	0.116	9.957	<0.001	0.010	0.000	0.277	28.014	<0.001
Education	−0.008	0.005	−0.017	−1.632	0.103	−0.069	0.004	−0.173	−18.533	<0.001
Occupation	0.029	0.011	0.025	2.513	0.012	0.030	0.006	0.032	4.777	<0.001
Marital status	0.004	0.006	0.005	0.609	0.542	0.003	0.003	0.006	0.781	0.435
Residence	0.012	0.010	0.010	1.171	0.241	0.021	0.008	0.017	2.624	0.009
Smoking	0.029	0.006	0.041	4.682	<0.001	0.035	0.007	0.033	4.896	<0.001
Alcohol	0.041	0.005	0.073	8.286	<0.001	−0.016	0.005	−0.023	−3.398	<0.001

*p* < 0.05; men: F = 48.505; R^2^ = 0.458; adjusted R^2^ = 0.209; women: F = 641.467; R^2^ = 0.534; adjusted R^2^ = 0.285.

**Table 4:** Odds ratio of MS for nutrition and dietary pattern.

Variables	Classification	Men MS, Odds Ratio		Women MS, Odds Ratio	
		Non-BLS	BLS	Non-BLS	BLS
Daily calories	reference	–	1.00	–	1.00
	G1, below RDI	1.00	0.99 (0.92–1.06)	1.00	1.02 (0.95–1.09)
	G2, above RDI	1.17 (1.02–2.19)	1.20 (1.04–1.76)	1.21 (1.09–2.19)	1.31 (1.12–2.50)
Breakfast	reference	–	1.00	–	1.00
	G1, low skipping	1.00	1.10 (0.93–1.30)	1.00	1.05 (0.91–1.21)
	G2, medium skipping	1.07 (0.94–1.21)	1.04 (0.91–1.19)	0.95 (0.84–1.08)	0.97 (0.85–1.69)
	G3, high skipping	1.19 (1.03–2.00)	1.24 (1.03–1.97)	1.10 (1.03–2.12)	1.11 (1.04–2.28)
Eating out	reference	–	1.00	–	1.00
	G1, low eating	1.00	0.98 (0.90–1.07)	1.00	0.89 (0.79–1.04)
	G2, medium eating	0.96 (0.87–1.06)	0.97 (0.88–1.08)	0.92 (0.82–1.09)	1.01 (0.89–1.52)
	G3, high eating	1.15 (1.01–1.86)	1.18 (0.98–1.39)	1.11 (1.10–1.94)	1.38 (0.84–2.57)
Nutritional education	reference	–	1.00	–	1.00
	G1, education	1.00	0.82 (0.65–1.04)	1.00	1.18 (0.83–1.97)
	G2, no education	1.17 (0.64–2.16)	1.11 (1.04–1.96)	1.18 (1.01–2.08)	1.20 (1.05–2.05)
Nutritional awareness	reference	–	1.00	–	1.00
	G1, awareness	1.00	1.01 (0.82–1.24)	1.00	1.02 (0.85–1.23)
	G2, no awareness	1.24 (1.02–1.91)	1.30 (1.07–1.77)	1.11 (1.02–1.92)	1.35 (1.14–2.59)
Dietary life condition	reference	–	1.00	–	1.00
	G1, sufficient	1.00	1.08 (0.92–1.26)	1.00	1.20 (0.90–1.60)
	G2, insufficient	0.98 (0.76–1.10)	0.93 (0.86–1.09)	0.91 (0.78–1.28)	0.94 (0.78–0.98)

G, group; RDI, reference dietary intake; BLS, basic livelihood security. Reference group: G1 of non-BLS. Adjusted variables, men: age, occupation, smoking, alcohol; women: age, education, occupation, residence, smoking, alcohol.

**Table 5:** Odds ratio of metabolic syndrome for activity type.

Variables	Classification	Men MS, Odds Ratio		Women MS, Odds Ratio	
		Non-BLS	BLS	Non-BLS	BLS
Walking	reference	–	1.00	–	1.00
	G1, 6–7/days	1.00	1.12 (0.91–1.39)	1.00	1.17 (0.96–1.42)
	G2, 3–5/days	1.19 (0.96–1.47)	0.89 (0.64–1.24)	1.21 (1.03–1.46)	1.20 (0.92–1.56)
	G3, 0–2/days	1.46 (1.17–1.83)	1.58 (1.27–1.96)	1.41 (1.08–1.82)	1.47 (1.22–1.78)
Strength training	reference	–	1.00	–	1.00
	G1, 4–7/days	1.00	1.29 (0.85–1.95)	1.00	1.35 (0.90–2.75)
	G2, 2–3/days	1.09 (0.69–1.72)	0.99 (0.62–1.57)	1.26 (1.05–2.14)	1.32 (0.89–2.88)
	G3, 0–1/day	1.41 (1.18–1.70)	1.57 (1.32–1.96)	2.11 (1.76–2.83)	2.16 (1.11–3.15)
MHA work	reference	–	1.00	–	1.00
	G1, hard labor	1.00	1.54 (0.96–2.45)	1.00	0.85 (0.53–1.38)
	G2, no hard labor	1.03 (0.92–1.15)	1.04 (0.93–1.17)	1.05 (0.91–1.21)	1.08 (0.94–1.25)
MHA leisure	reference	–	1.00	–	1.00
	G1, intense activity	1.00	1.18 (0.84–1.65)	1.00	1.20 (0.94–2.03)
	G2, no intense activity	1.26 (1.17–1.45)	1.28 (0.93–2.15)	1.18 (1.03–1.50)	1.24 (0.89–2.48)
Sedentary	reference	–	1.00	–	1.00
	G1, below 8.0 h	1.00	1.04 (0.85–1.27)	1.00	1.17 (0.97–1.41)
	G1, above 8.0 h	1.20 (1.09–1.39)	1.31 (1.09–1.69)	1.24 (1.07–1.42)	1.29 (0.96–1.58)

G, group; MHA, moderate to high activity; BLS, base livelihood security, MS, metabolic syndrome; BLS, basic livelihood security. Reference group: G1 of non-BLS. Adjusted variables, men: age, occupation, smoking, alcohol; women: age, education, occupation, residence, smoking, alcohol.

**Table 6:** Nutrient intake in groups.

Variables	Men		*t*	*p*	Women		*t*	*p*
	Non-BLS	BLS			Non-BLS	BLS		
Carbohydrate, g	323 ± 132.2	399.3 ± 179.2	−16.187	<0.001	320.6 ± 130.1	386.4 ± 171.1	−22.742	<0.001
Fat, g	42.5 ± 19.4	45.2 ± 17.3	−2.918	0.004	40.8 ± 15.0	43.9 ± 16.9	−5.355	<0.001
Unsaturated fatty acids, g	24.8 ± 8.6	23.3 ± 8.7	4.454	<0.001	24.2 ± 8.2	22.3 ± 8.0	7.109	<0.001
Saturated fatty acids, g	14.0 ± 5.1	13.6 ± 4.8	0.966	0.334	12.9 ± 5.6	13.6 ± 4.9	−2.924	0.003
Protein, g	79.0 ± 21.1	75.0 ± 26.4	4.299	<0.001	71.9 ± 26.5	74.6 ± 29.8	−4.615	<0.001
Dietary fiber, g	29.7 ± 10.5	27.6 ± 10.7	−5.177	0.003	30.5 ± 9.3	28.3 ± 10.9	−8.111	<0.001
Cholesterol, mg	220.7 ± 70.7	241.8 ± 78.3	−3.008	<0.001	217.8 ± 66.2	236.6 ± 75.5	−4.158	<0.001
Natrium, mg	3870.4 ± 200.1	4218.8 ± 242.6	−4.914	<0.001	3617 ± 196.4	3975.5 ± 252.1	−8.235	<0.001
Vitamin C, mg	68.9 ± 28.4	60.3 ± 28.9	2.814	0.005	77.2 ± 28.4	69.4 ± 20.0	4.092	<0.001

BLS, basic livelihood security.

## Data Availability

This study analyzed data released from government agencies: [https://knhanes.kdca.go.kr] (accessed on 20 January 2024).

## Supplementary Materials

The following supporting information can be downloaded at: https://www.mdpi.com/article/10.3390/nu16111677/s1. Table S1: Dietary Survey Questionnaire.
