# Supplementary material for: Prevalence of Metabolic Syndrome Based on Activity Type and Dietary Habits in Extremely Low-Income Individuals
Source: Nutrients. 2024 May 29;16(11):1677. doi: 10.3390/nu16111677 (PMC11175098; doi:10.3390/nu16111677)
Supplement: Supplementary file 1 [file nutrients-16-01677-s001.zip › nutrients-3025895-supplementary.pdf]

National Health and Nutrition Examination Survey 8th (2019-2021) Dietary Survey Table

승인번호 제 117002 호

The information contained in this survey form is kept confidential in accordance with Article 33 of the Statistics Act.

	district				residence		Household		household N.						
Subject											name		Gender	Male/Female	age	
respondent											name		Relationship with the subject			

Year Month Day staff name

Diet survey

*** Survey of eating habits**

1. The following questions are about the frequency of meals over the past year.

1-1. How many times a week have you eaten breakfast in the past year?

1. 5~7 times a week 2. 3~4 times a week 3. 1~2 times a week 4. Almost never (0 times a week)

1-2. How many times a week have you eaten lunch in the past year?

1.5~7 times a week 2. 3~4 times a week 3. 1~2 times a week 4. Almost never (0 times a week)

1-3. How many times a week have you had dinner in the past year?

1.5~7 times a week 2. 3~4 times a week 3. 1~2 times a week 4. Almost never (0 times a week)

2. The following questions ask whether you eat with other people when eating.

2-1. During the past year, when you had breakfast, did you usually eat it with other people?

1. Yes (=> Go to number 2-1-1) 2. No (=> Go to number 2-2) 3. Not applicable

2-1-1. Who did you usually eat with?

1. family 2. Other than family

2-2. During the past year, when you had lunch, did you usually eat with other people?

1. Yes (=> Go to number 2-2-1) 2. No (=> Go to number 2-3) 3.Not applicable

2-2-1. Who did you usually eat with?

1. family 2. Other than family

2-3. During the last year, when you had dinner, did you usually eat it with other people?

1. Yes (=> Go to No. 2-3-1) 2. No (=> Go to No. 3) 3. Not applicable

2-3-1. Who did you usually eat with?

1. family 2. Other than family

3. On average over the past year, eating out other than food cooked at home (meals (including delivery food, packaged food), school meals,

How often did you receive food provided by religious organizations, etc.?

1.More than twice a day 2. Once a day 3. 5-6 times a week 4. 3~4 times a week

5. 1~2 times a week 6. 1~3 times a month 7. Almost never (less than once a month)

*** Frequency of vegetable and fruit intake**

4. This is a question about the frequency of consumption of vegetables, mushrooms, and seaweed (seaweed, seaweed, etc.) over the past year.

4-1. On average, during the past year, how often have you consumed vegetables, mushrooms, and seaweed (seaweed, seaweed, etc.), including all vegetables?

This includes all side dishes, soups, stews, kimchi, and pickled vegetables.

1.More than 3 times a day	2. 5-6 times a week	3. 2~3 times a month
4. Twice a day	5. 2~4 times a week	6. Once a month
7. Once a day	8. Once a week	9. Almost never (less than once a month)

4-2. On average over the past year, excluding kimchi and pickled vegetables, mushrooms, and seaweed (seaweed, seaweed, etc.)

How often did you consume it all together? This also includes cases consumed as side dishes, soups, and stews.

1. More than 3 times a day	2. 5-6 times a week	3. 2~3 times a month
4. Twice a day	5. 2~4 times a week	6. Once a month
7. Once a day	8. Once a week	9. Almost never (less than once a month)

5. On average, how often have you consumed fruit over the past year?

1. More than 3 times a day	2. 5-6 times a week	3. 2~3 times a month
4. Twice a day	5. 2~4 times a week	6. Once a month
7. Once a day	8. Once a week	9. Almost never (less than once a month)

*** Dietary supplements**

6. Have you ever taken dietary supplements continuously for more than 2 weeks in the past year? 1. Yes 2. No

7. This is a question about the dietary supplements you are currently taking. 1.Not applicable (=> Go to number 8)

7-1. Type of product

1. Multivitamins and minerals<br>2. Vitamin C<br>3. Omega 3 fatty acid<br>4. Probiotics<br>5. Red ginseng<br>6. Calcium<br>7. Vitamin A & Lutein<br>8. Propolis<br>9. Vitamin D<br>10. Iron<br>11 Other vitamins and minerals<br>12. Others			

7-2. product name

7-3. Manufacturing company (distribution company)

***Dietary supplements**

7-4. Period of taking

1.Less than 1 month	1	1	1
2.1~3 months	2	2	2
3.4~6 months	3	3	3
4.7~11 months	4	4	4
5.More than 1 year	5	5	5

7-5. Frequency of taking

☐	☐	☐i	☐
1. Days	1	1	1
2. Week	2	2	2
3. Months	3	3	3

7-6. Whether or not it was taken 1 day before the survey

1.Yes	1	1	1
2.No	2	2	2

7-7. 1 dose

☐	☐	☐i	☐
1 Tablet (Tab)	1	1	1
2 Capsule	2	2	2
3 (Cap)	3	3	3
4 Pill	4	4	4
5 Bag or bat	5	5	5
6 Others ☐	6 ☐	6 ☐	6 ☐

* Nutritional Knowledge (Response only for elementary school students or older.]

8.Those who have received nutrition education and counseling provided at public health centers, district offices, community centers, welfare facilities, schools, hospitals, etc. in the past year Have you ever

1.Yes 2.No 3. Not application

9. The following is information about ‘Nutrition Labeling’. Do you know ‘Nutrition Labeling’?

1.Yes (=> Go to number 9-1) 2.No (=> Go to number 10) 3.Not applicable

9-1. Do you read the ‘nutrition label’ when buying or choosing processed foods?

1. Yes (=> Go to number 9-1-1) 2. No (=> Go to number 10)

9-1-1. What nutrients are you most interested in on the nutrition label?

1.Calories 2.Carbohydrates 3. Sugars 4. Protein 5. Fat
6.Saturated fat 7.Trans fat 8.cholesterol 9.Sodium 10.Others

9-1-2. Do nutrition labels influence your food choices? 1. Yes 2. No
